# Frequency independent, remotely reconfigurable passive coherent perfect absorber using conventional inkjet-printing technology

**DOI:** 10.1038/s41598-022-08665-4

**Published:** 2022-03-28

**Authors:** Stylianos D. Assimonis, Gabriel G. Machado, Vincent Fusco

**Affiliations:** Centre for Wireless Innovation (CWI), Institute of Electronics, Communications and Information Technology (ECIT), School of Electronics, Electrical Engineering and Computer Science (EEECS), Queen’s University Belfast, Belfast, BT3 9DT UK

**Keywords:** Engineering, Electrical and electronic engineering

## Abstract

This work presents a systematic theoretical analysis and experimental validation of a novel coherent absorber which is printed through conventional inkjet-printing technology. The new absorber consists of a single resistively loaded sheet printed on a conventional plastic sheet, resulting a low complexity and passive design. The low-cost and easily fabricated absorber is frequency independent, polarization insensitive, wide-angle and we demonstrate its absorbance reconfigurability using a remote illumination as a control signal. Theoretical, numerical and experimental results are in good agreement. Specifically, experimental results shown that near perfect absorption (i.e., $100\%$) can be achieved using a printed sheet of thickness $\lambda /215$.

## Introduction

Over the past decades, the researchers have focused on the field of electromagnetic absorbers. The first approach was the design which totally reflects an incidence electromagnetic wave in such a way that later destructively interfered with the reflected wave: Salisbury screen^1^ is the classic example of this technique, and also, the more recent electromagnetic bandgap structures (EBGs)^2^. Both the latter designs usually lead to geometries with dimensions comparably to the wavelength. Another approach, which has attracted significant attention over the last years, is the utilization of perfect metasurface absorbers (PMA): here the incident power is captured by the metasurface, which offers ideal impedance matching between free space and the lossy substrate and/or metallic parts of the absorber. Initial concepts have been realized on low-cost substrates (e.g., FR-4) by means of standard photo-lithographic techniques and proven to be very sub-wavelength, with a thickness of around one tenth of wavelength or less at their resonance frequency. In fact, the novel idea proposed by Landy et al.^3^ has led the prime focus in the analysis and fabrication of absorbing devices that operate in the microwave, terahertz, and even visible frequencies^4–22^. Furthermore, Sun et al. have demonstrated different techniques to control the deposition of inket droplets^23^ and the fabrication of high resolution patterns^24^ using a water-soluble sacrificial layer, which ultimately impacts on the performance of electronic materials.

On the other hand, the control of light with light is an engineering challenge that recently concerns the researchers^25–28^. The idea of a coherent perfect absorber (CPA) came up after the Huygens’ principle about the superposition of two light beams, which are traveling in a linear medium: waves pass through one other without mutual disturbance. In fact, the concept is to create disturbance by inserting a medium (e.g., dielectric slab, screen) along with traveling waves: under certain circumstances (i.e., waveform, wavelength and phase difference of the traveling waves) the medium will capture all, part or none of the incident power, providing control of the reflected/transmitted waves. Towards this direction, researchers have proposed CPA, which utilize dielectric slabs^25^, metasurfaces^27,29–36^ and conductive surfaces^37–42^. An inherent limitation in all such structures is bandwidth.

The contribution of this work is first the theoretical analysis of resistively loaded CPA geometries for normal and oblique incidence, and second, the proposal of a new way of producing resistively loaded CPAs using conventional inkjet printing technology. For the theoretical analysis, two models were utilized: the zero-thickness (ZT) resistive screen and the general slab (GS) model. Both the transmission line theory and full electromagnetic analysis were applied. The CPA models were optimized in terms of (perfect) absorbance and (full) remote wave reconfigurability. The energy dissipation was also studied. Theoretical analysis reveals that the proposed, purely resistive, zero-thickness CPA is frequency independent, polarization insensitive and wide-angle. Based on this analysis, a new CPA is proposed and fabricated. The latter is a simple resistively loaded sheet, printed on a plastic sheet, and to the best of the authors knowledge, conventional inkjet printing technology is utilized for the first time in the fabrication process of the CPAs. Specifically, the CPA was printed via a conventional inkjet printer by using conductive ink. Because the fabricated CPA is a resistive sheet with ultra low thickness (i.e., $\lambda /215$ at 10 GHz), its measured bandwidth is not unduly restricted. Please note that, although the proposed CPA was experimentally tested at microwave frequencies, it could be scaled to higher frequency region by the utilization of zero thickness graphene-based structures^28^.

## Results

**Figure 1:**
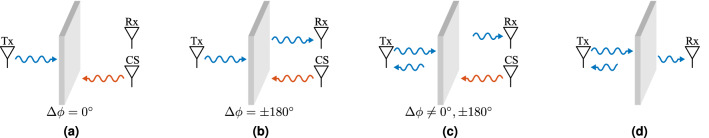
Concept illustration: the control signal (CS) controls the transmission/reflection through the absorbance. When the transmitted signal (Tx) is in-phase ($\Delta \phi =0^{\circ }$) with the CS, metasurface absorbs all the incident power (**a**), while when Tx and CS are out-phase ($\Delta \phi =\pm 180^{\circ }$) all the Tx is transmitted through the metasurface, which does not absorb of the incident power (**b**). When the phase difference between Tx and CS varies from $0^{\circ }$ to $\pm 180^{\circ }$ this also varies the absorbance by the metasurface, and thus, the transmitted/reflected power (**c**). Finally, when there is no CS, half of the power will absorbed by the absorber, while the rest will be equally reflected/transmitted (**d**).

The concept of the remotely reconfigurable and passive electromagnetic absorber is depicted in Fig. 1. The transmitted power from the transmitter (Tx) to the receiver (Rx) is controlled by the control signal (CS). Specifically, the remotely reconfigurable absorber is designed in order to permit zero transmission (maximum absorption, Fig. 1a) when Tx and CS are in-phase and maximum transmission (zero absorption, Fig. 1b) when these signals are out-phase Additionally, as the phase difference between Tx, CS varies from $0{^{\circ }}$. (in-phase) to $\pm 180{^{\circ }}$. (out-phase), the absorption, and hence, the transmitted/reflected power by the absorber also varies, as it will be shown (Fig. 1c). In the absence of CS (Fig. 1d), half of the incident power will be absorbed by the absorber, while the rest will be equally reflected and transmitted.

### Maximum power transfer

**Figure 2:**
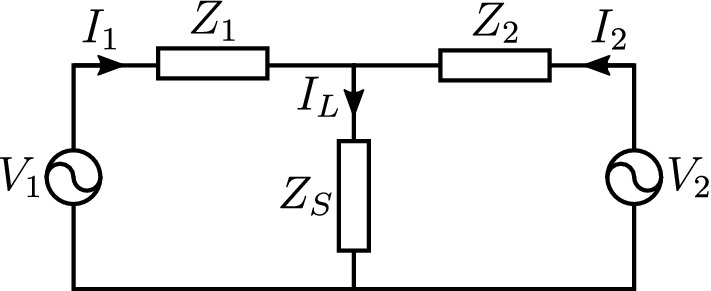
Two port circuit schematic: load $Z_S$ receives power from both sources with voltage $V_1$ and $V_2$.

The two port schematic for the purely resistive circuit is depicted in Fig. 2. Based on Kirchhoff’s law for the currents and voltages the transferred power $P_L$ to the load $Z_L$ is given by,

1 $$\begin{aligned} P_L = I_L^2 Z_S = \left( \frac{V_1 Z_2 + V_2 Z_1}{Z_1 Z_2 + Z_1 Z_S + Z_2 Z_S} \right) ^2 Z_S. \end{aligned}$$

Assuming that the two sources represent two plane waves propagating in the free space, normally incident on the screen, then

2 $$\begin{aligned} Z_1 = Z_2 = \eta _0, \end{aligned}$$

where $\eta _0 = 120\pi$ $\Omega$ is the characteristic (intrinsic) impedance of the free space, is through Eq. (1),

3 $$\begin{aligned} P_L = \left( \frac{V_1 + V_2}{\eta _0 + 2 Z_S} \right) ^2 Z_S. \end{aligned}$$

For $V_1 = V_2 = 1$ (i.e., in-phase) the power delivered to the load is maximized when

4 $$\begin{aligned} Z_S = \frac{\eta _0}{2} \end{aligned}$$

and it is given by

5 $$\begin{aligned} P_{L,\mathrm {max}} = \frac{ 1 }{ 2 \eta _0 }. \end{aligned}$$

For $V_1 = -V_2 = 1$ (i.e., out-phase) the power delivered to the load reduces to zero.

### Zero thickness model

#### Transmission line theory

**Figure 3:**
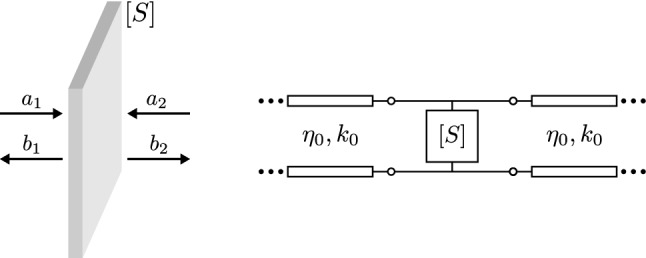
(left) General surface, which is represented by its equivalent scattering matrix, $\left[ S \right]$, is simultaneously illuminated by two signals, $a_1$ and $a_2$. The part of the incident power, which is not reflected or transmitted because of the presence of the surface, it is dissipated into the latter, i.e., it is absorbed by the surface. It is shown that, by adjusting the phase difference between the $a_1$ and $a_2$, it is possible to remotely adjust the absorbance of the surface, and, thus, to customise the transmitted/reflected power. (right) The equivalent circuit schematic of the passive reconfigurable electromagnetic absorber: the infinite transmission lines with wave impedance and number $\eta _0$ and $k_0$, respectively, represents the free space.

Figure 3 depicts a general surface, which is simultaneously illuminated by two signals, $a_1$ and $a_2$ (i.e., “two-port scatterer”) and its equivalent circuit schematic. The surface is represented by the scattering matrix, $\left[ S \right]$, and is surrounded by free space: infinite transmission lines with wave impedance and number $\eta _0$ and $k_0 = 2\pi /\lambda _0$, respectively, where $\lambda _0$ is the wavelength in free space, surround the surface. For a two-port scatterer, the joint absorption is given by^28^,

6 $$\begin{aligned} A = 1 - \frac{\left| b_1 \right| ^2 + \left| b_2\right| ^2 }{\left| a_1 \right| ^2 + \left| a_2 \right| ^2}, \end{aligned}$$

where, based on the scattering matrix of the surface is,

7a $$\begin{aligned} b_1&= S_{11} a_1 + S_{12} a_2, \end{aligned}$$

7b $$\begin{aligned} b_2&= S_{21} a_1 + S_{22} a_2. \end{aligned}$$

The two input signals could be in- or out-of-phase, while in general they have phase difference of $\Delta \phi$, and hence,

8a $$\begin{aligned} a_1&= 1, \end{aligned}$$

8b $$\begin{aligned} a_2&= e^{j \Delta \phi }, \end{aligned}$$

where, $\Delta \phi$ varies from $-180{^{\circ }}$ to $180{^{\circ }}$.

Next, it is assumed that the surface is symmetrical (i.e., $S_{11}=S_{22}$) and reciprocal (i.e., $S_{12}=S_{21}$)^43^, while presenting maximum absorbance (i.e., $A=1$) for $a_1=a_2=1$ (in-phase) and minimum (i.e., $A=0$) for $a_1=-a_2=1$ (out-of-phase). Thus, based on Eqs. (6)–(8) it is apparent that,

9 $$\begin{aligned} \left[ S \right] = \begin{bmatrix} S_{11} &{} S_{12} \\ S_{21} &{} S_{22} \end{bmatrix} = \begin{bmatrix} -1/2 &{} 1/2 \\ 1/2 &{} -1/2 \end{bmatrix} \end{aligned}$$

and the absorbance through Eqs. (6) and (9) is given by,

10 $$\begin{aligned} A = 1 - \frac{1}{4} \left| 1 - e^{j\Delta \phi } \right| ^2. \end{aligned}$$

**Figure 4:**
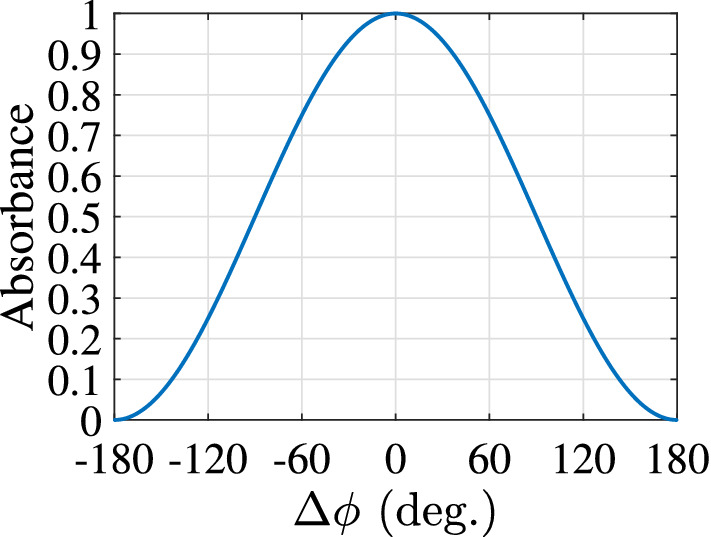
The joint absorption of the proposed passive electromagnetic absorber with the specific scattering matrix given by Eq. (9): when it is illuminated by two signals of the same frequency and with the same amplitude and phase difference of $\Delta \phi$.

The estimated absorption versus $\Delta \phi$ is depicted in Fig. 4: for $\Delta \phi =0{^{\circ }}$. (in-phase) the absorption is maximised and the total power from signals $a_1$, $a_2$ is captured by the surface, while for $\Delta \phi =\pm 180{^{\circ }}$. (out-of-phase) the absorption is minimised and the signals $a_1$, $a_2$ propagate through the surface without losses. In all the other cases, where $\Delta \phi \ne 0{^{\circ }}$. or $\Delta \phi \ne \pm 180{^{\circ }}$, part of the incident power is absorbed and the rest is reflected or transmitted accordingly. On the other hand, where there is only one signal (e.g., only signal $a_1$), the half of its power dissipates into the surface, since based on Eq. (6) is $A = 0.5$, while the rest of its power is equally reflected and transmitted, since based on Eq. (9) is $S_{11} = -0.5$ and $S_{21} = 0.5$, respectively. Based on the latter analysis, it is shown that, by using a control signal ($a_2$) it is possible to remotely adjust the absorbance of a surface, and thus, to customise the transmitted or the reflected power: when the incident signals $a_1$, $a_2$ are in- (out-of-) phase perfect (no) absorption occurs, while in the absence of $a_2$, $A=0.5$.

Hence, remotely re-configurable passive electromagnetic absorbers are possible to be designed. It is noted that the above analysis took place for normal incident signals and the results are frequency independent due to the ZT surface.

Next, it is assumed that the surface is an ohmic sheet with surface resistance of $Z_{\mathrm {S}}$. Based on the transmission line theory^43^, the *ABCD* parameters of the latter two-port circuit is given by,

11 $$\begin{aligned} \begin{bmatrix} A &{} B \\ C &{} D \end{bmatrix} = \begin{bmatrix} 1 &{} 0 \\ {1}/{Z_{S}} &{} 1 \end{bmatrix}, \end{aligned}$$

and hence, since the slab is surround by free space, the equivalent scattering matrix is given by,

12 $$\begin{aligned} \left[ S \right] = \frac{1}{2 Z_{S}+\eta _0} \begin{bmatrix} -\eta _0 &{} 2 Z_{S} \\ 2 Z_{S} &{} -\eta _0 \end{bmatrix}. \end{aligned}$$

Applying Eq. (9) in (12) results in

13 $$\begin{aligned} Z_{S} = \frac{\eta _0}{2}. \end{aligned}$$

The latter equation is in alignment with Eq. (4).

Moreover, Eqs. (4) and (13) reveal that the new surface is in some respects equivalent to the Salisbury screen^1^, where a resistively loaded screen with surface resistance of $\eta _0$ is placed at distance of $\lambda _0/4$ from a ground plane. On the latter surface, the reflected signal by the ground plane is suppressed by the incident signal because they have phase difference of $180{^{\circ }}$. (out-of-phase), and thus, there is no reflection Here, the proposed ZT coherent absorber has no ground plane, however, on the screen with surface resistance of $\eta _0 / 2$ the incident signal $a_1$ is suppressed by the also incident signal $a_2$ (control signal) because of their phase difference. The total incident power is not reflected/transmitted as an electromagnetic wave (i.e., it does not propagate either backward of forward) but is transformed into surface current, which is dissipated in the resistively loaded screen: the incident power is absorbed by the surface. Hence, in this reconfigurable but passive electromagnetic absorber the ground plane is “virtual” and could effectively “appear” or “disappear” depending on the phase difference of the signals $a_1$ and $a_2$. However, because of the physical absence of the ground plane and the use of a zero-thickness purely resistive sheet (i.e., ZT-model), the new coherent perfect absorber is frequency independent.

#### Electromagnetic analysis

**Figure 5:**
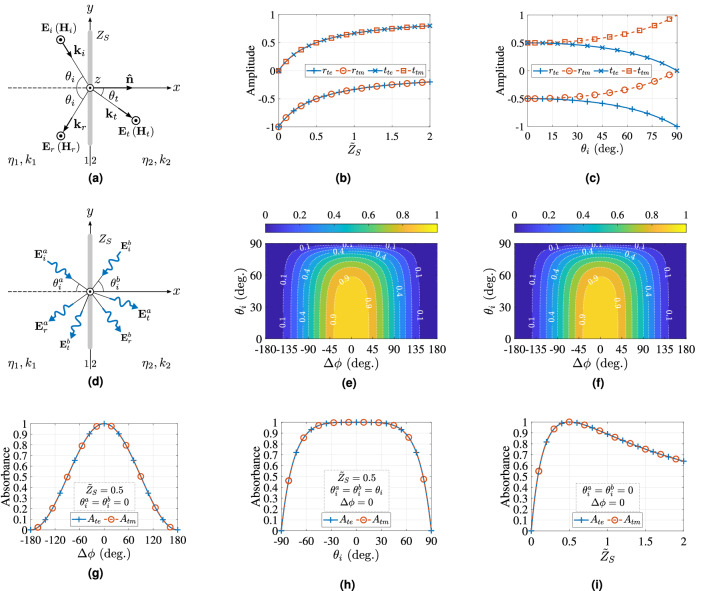
Oblique incidence representation for TE (TM) -polarization on a resistively loaded screen with surface impedance of $Z_S$ (**a**). The reflection and transmission coefficient on the latter screen for both polarizations versus the normalized surface impedance, i.e., ${\tilde{Z}}_S = Z_S/\eta _0$ (**b**) and versus angle of incidence (**c**). The screen is illuminated by two plane waves ${\mathbf {E}}_i^a$ and ${\mathbf {E}}_i^b$ with angle of incidence of $\theta _i^a$ and $\theta _i^b$, respectively, and with phase difference of $\Delta \phi$ (**d**). Assuming that $\theta _i^a = \theta _i^b = \theta _i$, the joint absorbance versus $\Delta \phi$ and $\theta _i$ is depicted for TE (**e**) and TM (**f**) polarization. Also depicted is the joint absorbance for the special cases when ${\tilde{Z}}_S = 0.5$ and $\theta _i^a = \theta _i^b = 0{^{\circ }}$ versus $\Delta \phi$ (**g**) or when ${\tilde{Z}}_S = 0.5$, $\theta _i^a = \theta _i^b = \theta _i$ and $\Delta \phi =0{^{\circ }}$ versus $\theta _i$ (**h**) or when $\theta _i^a = \theta _i^b = 0{^{\circ }}$ and $\Delta \phi =0{^{\circ }}$ versus ${\tilde{Z}}_S$ (**i**). Based on the above analysis it is evident that the proposed absorber presents absorbance higher than 0.9 for $-58.7{^{\circ }} \le \theta _i \le 58.7{^{\circ }}$ and $-36.7{^{\circ }} \le \Delta \phi \le 36.7{^{\circ }}$, and thus it is wide-angle, polarization insensitive and robust to the phase-offset between the transmitted and the control signal.

Figure 5a depicts the oblique incidence on a zero-thickness resistively loaded sheet with surface impedance of $Z_S$. The latter lies on the interface $S_m$ of two different bulk media (1 and 2) with relative permittivity and permeability of $\varepsilon _{r,1(2)}$ and $\mu _{r,1(2)}$ respectively. Thus, medium 1(2) has intrinsic impedance $\eta _{1(2)} = \eta _0 \sqrt{\mu _{r,1(2)}/\varepsilon _{r,1(2)}}$ and wavenumber $k_{1(2)}= 2\pi /\lambda _0 \sqrt{\mu _{r,1(2)} \varepsilon _{r,1(2)}}$. It is noted that the $Z_S$ is defined as the ratio of the transverse components of the electric and magnetic fields on the $S_m$. The Fresnel Equations (based on the boundary conditions) for that case is^44^

14 $$\begin{aligned} \left. {\hat{\mathbf {n}}} \times \left( {{\mathbf {E}}}_i + {{\mathbf {E}}}_r \right) \right| _{S_m^{-}}&= \left. {\hat{\mathbf {n}}} \times {{\mathbf {E}}}_t \right| _{S_m^{+}} \end{aligned}$$

15 $$\begin{aligned} \left. {\hat{\mathbf {n}}} \times \left( {{\mathbf {H}}}_i + {{\mathbf {H}}}_r \right) \right| _{S_m^{-}}&= \left. {\hat{\mathbf {n}}} \times {{\mathbf {H}}}_t \right| _{S_m^{+}} - {{\mathbf {J}}}_s \end{aligned}$$

where ${\hat{\mathbf {n}}}$ is the unit-vector perpendicular to the $S_m$ with direction from medium 2 to 1 and ${\mathbf {J}}_s$ is the surface current distribution on the $S_m$. Based on the Ohm’s law, for the latter is

16 $$\begin{aligned} {{\mathbf {J}}}_s = \frac{1 }{Z_s} {{\mathbf {E}}}_s, \end{aligned}$$

where ${{\mathbf {E}}}_s$ is the tangential component of the electric field to the $S_m$: it is noted that the surface current is always in parallel to ${{\mathbf {E}}}_s$. Specifically, for the TE-, TM-polarization is

17 $$\begin{aligned} {{{\mathbf {E}}}_s^{TE}}&= {\dot{E}}_i \, {\hat{\mathbf {z}}}, \end{aligned}$$

18 $$\begin{aligned} {{{\mathbf {E}}}_s^{TM}}&= {\dot{E}}_i \cos \theta {}_i \, {\hat{\mathbf {y}}} \end{aligned}$$

respectively, where, $\theta _i$ is the angle of incidence. The relation between the magnetic and electric field of the incident and reflected plane wave is

19 $$\begin{aligned} {{\mathbf {H}}}_{i/r } = \frac{{\hat{\mathbf {n}}}_{i/r} \times {{\mathbf {E}}}_{i/r}}{\eta _1} \end{aligned}$$

respectively, while for the transmitted wave is

20 $$\begin{aligned} {{\mathbf {H}}}_{t} = \frac{{\hat{\mathbf {n}}}_{t} \times {{\mathbf {E}}}_{t}}{\eta _2}, \end{aligned}$$

where the ${\hat{\mathbf {n}}}_{i/r/t}$ is the normalized wave vector for the incident, reflected and transmitted wave, respectively, and $\eta _{1,2}$ is the wave impedance for the medium 1, 2, respectively, as mentioned. Using Eqs. (19) and (20) we can write Eq. (15) as

21 $$\begin{aligned} \left. \frac{ {\hat{\mathbf {n}}} \times \left( {\hat{\mathbf {n}}}_{i} \times {{\mathbf {E}}}_{i} + {\hat{\mathbf {n}}}_{r} \times {{\mathbf {E}}}_{r} \right) }{\eta _1} \right| _{S_m^{-}}&= \left. \frac{ {\hat{\mathbf {n}}} \times \left( {\hat{\mathbf {n}}}_{t} \times {{\mathbf {E}}}_{t} \right) }{\eta _2} \right| _{S_m^{+}} - {{\mathbf {J}}}_s \end{aligned}$$

The reflection and transmission coefficients for the TE- and TM-polarization are calculated by solving the system of Eqs. (14) and (21) using Eqs. (16), (17) and (18) and are given by,

22a $$\begin{aligned} r_{{te}}:&= \frac{{\dot{E}}_r}{{\dot{E}}_i} = \frac{ \eta _2\cos \theta _i - \eta _1\cos \theta _t - q }{ \eta _2\cos \theta _i + \eta _1\cos \theta _t + q } \end{aligned}$$

22b $$\begin{aligned} {t_{{te}}}:&= \frac{{\dot{E}}_t}{{\dot{E}}_i} = \frac{ 2 \eta _2 \cos \theta _i }{ \eta _2\cos \theta _i + \eta _1\cos \theta _t + q } \end{aligned}$$

22c $$\begin{aligned} r_{{tm}}:&= \frac{{\dot{E}}_r}{{\dot{E}}_i} = \frac{ \eta _2\cos \theta _t - \eta _1\cos \theta _i - q \cos \theta _i \cos \theta _t }{ \eta _2\cos \theta _t + \eta _1\cos \theta _i + q \cos \theta _i \cos \theta _t } \end{aligned}$$

22d $$\begin{aligned} t_{{tm}}:&= \frac{{\dot{E}}_t}{{\dot{E}}_i} = \frac{ 2 \eta _2 \cos \theta _i }{ \eta _2\cos \theta _t + \eta _1\cos \theta _i + q \cos \theta _i \cos \theta _t } \end{aligned}$$

where, $q = \eta _1 \eta _2 / Z_s$ and $\theta _t$ is the angle of transmission (refraction) and

23 $$\begin{aligned} \sin \theta _t = \frac{k_1}{k_2} \sin \theta _i, \end{aligned}$$

where, ${\dot{E}}_i$, ${\dot{E}}_r$, ${\dot{E}}_t$ denotes the complex amplitude of the incident, reflected and transmitted (refracted) wave, respectively. It is obvious that

24a $$\begin{aligned} t_{{te}}&= r_{{te}} + 1 \end{aligned}$$

24b $$\begin{aligned} \frac{\cos \theta _t}{\cos \theta _i}t_{{tm}}&= r_{{tm}} + 1. \end{aligned}$$

The above equations are in alignment with the analysis in^45^ of a two-dimensional crystal. It is assumed that the resistively loaded surface is surrounded by free space, and thus,

25a $$\begin{aligned} \eta _1&=\eta _2=\eta _0 \end{aligned}$$

25b $$\begin{aligned} k_1&=k_2=k_0. \end{aligned}$$

From Eqs. (23) and (25a, 25b) it turns

26 $$\begin{aligned} \theta _i = \theta _t. \end{aligned}$$

Figure 5b,c depict the reflection and transmission coefficients of the resistively loaded screen for both TE- and TM-polarization versus the normalized surface impedance ${\tilde{Z}}_S = {Z}_S/\eta _0$ for normal incidence (i.e., $\theta _i =0{^{\circ }}$) and versus the angle of incidence $\theta _i$ for ${\tilde{Z}}_S = 0.5$, respectively: it is noted that $r_{te} = r_{tm} = -0.5$ and $t_{te} = t_{tm} = 0.5$ for ${\tilde{Z}}_S = 0.5$ and $\theta _i = 0{^{\circ }}$.

Next, it is assumed that the zero-thickness, resistivity loaded sheet is simultaneously illuminated by two planes waves, the ${{\mathbf {E}}}^a_i$, which impinges with an angle of $\theta ^a_i$ and the ${{\mathbf {E}}}^b_i$, which impinges with an angle of $\theta ^b_i$, as shown in Fig. 5d. The joint absorption is now given by

27 $$\begin{aligned} A = 1 - \frac{ \left| {\dot{E}}^{a}_{r} + {\dot{E}}^{b}_{t} \right| ^2 + \left| {\dot{E}}^{b}_{r} + {\dot{E}}_{a}^{t} \right| ^2 }{\left| {E}_{a}^{i}\right| ^2 + \left| {E}^{b}_{i}\right| ^2}, \end{aligned}$$

which is in accordance with Eq. (6).

Again the signals ${\dot{E}}^{a}_{i}$, ${\dot{E}}^{b}_{i}$ could be in- or out-phase, and hence it is assumed,

28a $$\begin{aligned} {\dot{E}}^{a}_{i}&= 1, \end{aligned}$$

28b $$\begin{aligned} {\dot{E}}^{b}_{i}&= e^{j \Delta \phi } , \end{aligned}$$

similar to Eq. (8).

For normal incidence (i.e., $\theta ^a_i = \theta ^b_i = 0{^{\circ }}$) the reflection and transmission coefficients are now through Eq. (22),

29a $$\begin{aligned} r&= -\frac{1}{2 {\tilde{Z}}_S + 1} \end{aligned}$$

29b $$\begin{aligned} t&= \frac{2 {\tilde{Z}}_S}{2 {\tilde{Z}}_S + 1}, \end{aligned}$$

and thus, the absorption is now

30 $$\begin{aligned} A = 1 - \frac{1}{2} \left( \left| r + t e^{j \Delta \phi } \right| ^2 + \left| t + r e^{j \Delta \phi } \right| ^2 \right) . \end{aligned}$$

For the case of in-phase signals ($\Delta \phi =0{^{\circ }}$) the absorbance is

31 $$\begin{aligned} A = 1 - \left| \frac{2 {\tilde{Z}}_S - 1}{2 {\tilde{Z}}_S + 1} \right| ^2, \end{aligned}$$

which is maximized for

32 $$\begin{aligned} {\tilde{Z}}_S = \frac{1}{2} \end{aligned}$$

which is in alignment to Eqs. (4) and (13), as expected.

#### Incidence at the same angle

It is assumed that the two waves impinge to the resistively loaded screen of ${\tilde{Z}}_S = 0.5$ with the same angle of incidence, i.e., $\theta ^a_i = \theta ^b_i = \theta _i$ (Fig. 5d). The joint absorbance for that case and after algebraic manipulation of Eqs. (22), (26), (27) and (28) is for TE- and TM-polarization,

33 $$\begin{aligned} A = 1 - \frac{ \left| 1 - e^{ j \Delta \phi } \cos \theta _i \right| ^2 + \left| \cos \theta _i - e^{ j \Delta \phi } \right| ^2 }{2\left| \cos \theta _i + 1 \right| ^2}. \end{aligned}$$

Figure 5e,f depict the joint absorbance: $A \ge 0.9$ for $-58.7{^{\circ }} \le \theta _i \le 58.7{^{\circ }}$ and for $-36.7{^{\circ }} \le \Delta \phi \le 36.7{^{\circ }}$ for both polarizations. The latter reveals that the proposed absorber is wide-angle, polarization insensitive and robust to the phase-offset between the transmitted and the control signal.

For the special case of normal incidence (i.e., $\theta _i=0{^{\circ }}$) the joint absorbance versus $\Delta \phi$ based on Eq. (33) is

34 $$\begin{aligned} A = 1 - \frac{1}{4} \left| 1 - e^{j\Delta \phi } \right| ^2, \end{aligned}$$

which is in accordance to Eq. (10), while Fig. 5g: depicts the results.

Figure 5h depicts the joint absorbance for oblique incidence and with in-phase waves (i.e., $\Delta \phi =0{^{\circ }}$): in that case is

35 $$\begin{aligned} A = 1 - \left| \frac{\cos \theta _i - 1}{\cos \theta _i + 1 } \right| ^2. \end{aligned}$$

It is obvious that it is $A \ge 0.9$ when $-58.7{^{\circ }} \le \theta _i \le 58.7{^{\circ }}$, as expected. Finally, Fig. 5i depicts the joint absorbance for normal incidence and for in-phase waves based on Eq. (31): *A* is maximized when ${\tilde{Z}}_S = 0.5$, as expected.

#### Incidence at different angle

**Figure 6:**
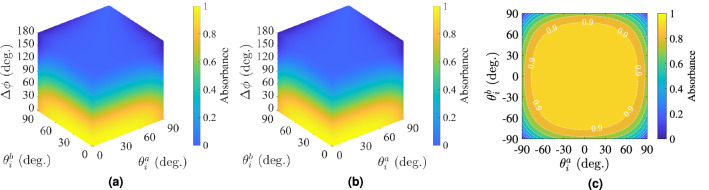
The joint absorbance of the proposed absorber versus angle of incidence $\theta _i^a,~\theta _i^b$ and phase difference $\Delta \phi$ between the two signals ${{\mathbf {E}}}^a_i,~ {{\mathbf {E}}}^b_i$ for the TE- (**a**) and TM-polarization (**b**): it is observed that the resistively loaded screen presents absorbance higher than 0.9 when $-77{^{\circ }} \le \theta _i^a ,~\theta _i^b \le 77{^{\circ }}$ and $-36.7{^{\circ }} \le \Delta \phi \le 36.7{^{\circ }}$. The case where the two plane waves are in-phase is presented in (**c**).

It is assumed that the two waves impinge to the resistively loaded screen of ${\tilde{Z}}_S = 0.5$ with different angle of incidence, i.e., $\theta ^a_i \ne \theta ^b_i$ (Fig. 5d). The joint absorbance for that case is now given by,

36a $$\begin{aligned} A_{te}&= 1 - \frac{1}{2} \left| \frac{1}{1+\cos \theta _i^a} - \frac{\cos \theta _i^b}{1+\cos \theta _i^b} e^{j \Delta \phi } \right| ^2 - \frac{1}{2} \left| \frac{1}{1+\cos \theta _i^b} e^{j \Delta \phi } - \frac{\cos \theta _i^a}{1+\cos \theta _i^a} \right| ^2 \end{aligned}$$

36b $$\begin{aligned} A_{tm}&= 1 - \frac{\left| \left( 1+\cos \theta _i^b \right) - \left( 1+\cos \theta _i^a \right) \cos \theta _i^b e^{\Delta \phi } \right| ^2 }{2 \left( 1+\cos \theta _i^a \right) ^2 \left( 1+\cos \theta _i^b \right) ^2} - \frac{ \left| \left( 1+\cos \theta _i^a \right) e^{\Delta \phi } - \left( 1+\cos \theta _i^b \right) \cos \theta _i^a \right| ^2 }{2 \left( 1+\cos \theta _i^a \right) ^2 \left( 1+\cos \theta _i^b \right) ^2} \end{aligned}$$

for the TE- and TM-polarization, respectively, and it is depicted in Fig. 6a,b versus angles of incidence $\theta ^a_i$, $\theta ^b_i$ and the phase difference $\Delta \phi$ of the two incident signals for both polarizations, TE and TM, respectively. It is evident that as $\theta _i^a, ~\theta _i^b$ varies from $-77{^{\circ }}$ to $77{^{\circ }}$ and $\Delta \phi$ varies from $-36.7{^{\circ }}$ to $33.7{^{\circ }}$, the joint absorbance remains higher than 0.9. In particular, for the case where the two plane waves are in-phase, the joint absorbance is given by

37 $$\begin{aligned} A = 1 - \left| \frac{\cos \theta _i^a \, \cos \theta _i^b - 1}{\left( \cos \theta _i^a + 1 \right) \left( \cos \theta _i^b + 1 \right) } \right| ^2, \end{aligned}$$

for both polarizations. Equation (37) also gives the relation between the $\theta ^a_i,~\theta ^b_i$ when *A* is higher than a specific threshold, i.e., $A \ge A_{0}$: the results are depicted in Fig. 6c.

The latter analysis again reveals that the proposed CPA is robust to the angle-of-incidence mismatch and to the phase offset between the transmitted and the control signal (as depicted in Fig. 1).

**Figure 7:**
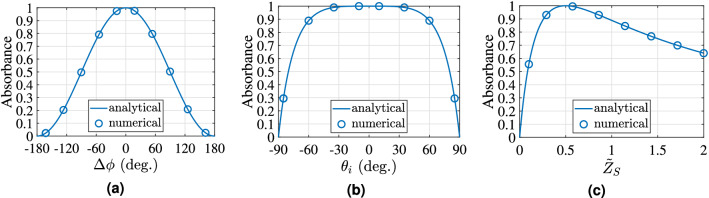
Analytical versus numerical results for the frequency independent screen. The joint absorbance versus $\Delta \phi$ (**a**), angle of incidence (**b**) and surface resistance (**c**) was estimated through Eqs. (34) and (35) and (31), respectively, for the analytical results, while for the numerical results was estimated through Eqs. (6), (7) and the simulated *S* parameters: a very good agreement is observed.

Next, the ZT-model was tested through numerical analysis. A resistively loaded screen was simulated by using the commercial CST Microwave Studio (MWS) electromagnetic solver^46^. The screen was modelled as ohmic sheet with zero thickness and surface resistance of $\eta _0/2 = 60\pi$ ${\mathrm {\Omega /sq}}$. Assuming that the surface is infinite, the geometry was simulated by using Floquet periodic boundary conditions, while a unit-cell was assumed a sheet with dimensions $\lambda /2 \times \lambda /2$ at 10 GHz (please note that the size is irrelevant since a homogeneous *ohmic sheet*^46^ is used as unit-cell), effectively transforming the resistive surface, located in the centre of the boundary box, into an infinite sheet, excited from two periodic ports, which were used as the source for a plane wave excitation, and the reference plane moved to the location of the sheet (i.e., reflection and transmission coefficients de-embedding). Based on theoretical analysis of the ZT-model, the resistively loaded screen is frequency independent, however, without lack of generality, 10 GHz was chosen as centre frequency (i.e., $\lambda \approx 30$ mm). The unit-cell terminates into two periodic ports and the reflection/transmission coefficients (i.e., *S* parameters) of the resulting resistive screen was estimated. Then, the joint absorbance was calculated based on the simulated *S* parameters and via the Eqs. (6) and (7). Figure 7 depicts the analytical versus numerical results for the joint absorbance versus $\Delta \phi$ (when $Z_s =\eta _0/2$ and $\theta _i^a=\theta _t^b=0{^{\circ }}$), angle of incidence (when $Z_s =\eta _0/2$, $\Delta \phi =0{^{\circ }}$ and $\theta _i=\theta _t=\theta _i$.) and surface resistance (when $\Delta \phi =0{^{\circ }}$ and $\theta _i=\theta _t=0{^{\circ }}$): it is evident that the latter agree very well.

**Figure 8:**
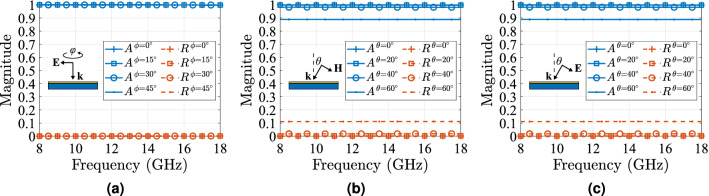
Numerical results for the proposed CPA to demonstrate its polarization stability. The joint absorbance, *A*, and reflectance, *R*, versus frequency for normal incidence as azimuthal angle $\varphi$ varies from $0{^{\circ }}$ to $45{^{\circ }}$ (**a**), and for oblique incidence as polar angle $\theta=\theta_i$ varies from $0{^{\circ}}$ to $60{^{\circ }}$ for a plane wave TE- (**b**) and TM-polarised (**c**): the proposed absorber is frequency independent, polarization insensitive and presents a high angular stability.

Furthermore, to demonstrate the CPAs capabilities of being polarization insensitive frequency independent and wide-angle, we have carried out a simulation in CST MWS to obtain absorbance, *A*, and reflectance, *R*, across a frequency range of 8–18 GHz and the results are depicted in Fig. 8. For this scenario, the boundary conditions were maintained, whilst for normal incidence the electric component of the incident plane wave was rotated by the azimuthal angle $\varphi$ from 0 to $45{^{\circ }}$ (Fig. 8a): *A* and *R* remained unchanged over this variation, as expected based on the theoretical analysis. For oblique incidence the TE- and TM-polarisation were tested, as depicted in Fig. 8b,c, respectively. It is evident that, firstly, the CPA’s *A* and *R* is independent of the polarisation, secondly, *A* remains higher that $90\%$ for angle of incidence up to $\theta =\theta _i= 60{^{\circ }}$ (this is in perfect agreement with Fig. 7b), and thirdly, both *A* and *R* are independent of frequency for every case under test. Based on the above the proposed CPA is wide-angle, polarisation insensitive and frequency independent. For each case under test, *A* and *R* are independent of frequency. Based, thus, the CPA is wide-angle. as expected based on the theoretical analysis.

#### Where is the energy going?

In any electromagnetic absorber, the captured energy dissipates in the dielectric and metallic parts. For the dielectric parts the power losses is given by^44^

38 $$\begin{aligned} P_d = \pi \varepsilon f \tan \delta \iiint \limits _{V_d} \left\| {\mathbf {E}} \right\| ^2 {\mathrm {d}}V \end{aligned}$$

where $\varepsilon$, $\tan \delta$ and $V_d$ is the substrate’s permittivity, tangential losses and volume, respectively, while *E* is the electric field and *f* the frequency of the propagating plane wave within the medium $V_d$. Similarly, for the conducting metallic parts,

39 $$\begin{aligned} P_m = \frac{1}{2} {\mathrm {Re}} \left\{ \sqrt{ \frac{\pi \mu f}{\sigma }} \right\} \iint \limits _{S_m} \left\| {\mathbf {H}}_t \right\| ^2 {\mathrm {d}}S \end{aligned}$$

where, $\mu$, $\sigma$ and $S_m$, is the conductor’s permeability, conductivity and surface, respectively, while $H_t$ is the magnetic field on the $S_m$ (tangent to the surface) of the propagating plane wave.

For the case of the resistively loaded screen with surface resistance of $Z_L$ the absorbed energy dissipates only on the metallic (i.e., resistive) parts (i.e., $P_d=0$) and now is

40 $$\begin{aligned} P_m = \frac{1}{2} Z_L \iint \limits _{S_m} \left\| {\mathbf {H}}_t \right\| ^2 {\mathrm {d}}S, \end{aligned}$$

since

41 $$\begin{aligned} \sigma = \frac{1}{Z_L d}, \end{aligned}$$

and *d* is the thickness of the screen, which is assumed to be equal to the skin depth, and thus,

42 $$\begin{aligned} d \approx \sqrt{\frac{1}{\mu \sigma \pi f}}. \end{aligned}$$

For normal incidence (Fig. 5a) and free space is

43a $$\begin{aligned} {\mathbf {H}}^a_i&= \dot{H^a_i} \, {\hat{\mathbf {z}}} \, e^{ -j k_0 {x} } = \frac{\dot{E^a_i}}{\eta _0} \, {\hat{\mathbf {z}}} \, e^{ -j {k_0 x} } \end{aligned}$$

43b $$\begin{aligned} {\mathbf {H}}^b_i&= \dot{H^b_i} \, {\hat{\mathbf {z}}} \, e^{ +j k_0 {x} } = \frac{\dot{E^b_i}}{\eta _0} \, {\hat{\mathbf {z}}} \, e^{ +j {k_0 x} }. \end{aligned}$$

Thus, the total tangential magnetic field on the resistively loaded surface ($x=0$) is

44 $$\begin{aligned} \left\| {\mathbf {H}}_t \right\| ^2 = \left| \frac{ \dot{E^a_i}+\dot{E^b_i} }{ \eta _0 } \right| ^2 \end{aligned}$$

Substituting Eqs. (13), (44) and (28) into (40), the absorbed power by the resistive screen is given by,

45 $$\begin{aligned} P_m = \frac{1}{4} \frac{\left| 1 + e^{j\Delta \phi } \right| ^2}{\eta _0 } S_m. \end{aligned}$$

On the other hand, the power input into the resistive screen can be estimated through the power density (time average) of the two incident plane waves, and thus is

46 $$\begin{aligned} \begin{aligned} P_{in} = \frac{1}{2} \left[ {\mathrm {Re}}\lbrace {\mathbf {E}}_a^i \times {{\mathbf {H}}}_a^{i,*} \rbrace + {\mathrm {Re}}\lbrace {\mathbf {E}}_b^i \times {{\mathbf {H}}}_b^{i,*} \rbrace \right] S_m = \frac{\left| {\dot{E}}_{a}^{i} \right| ^2 + \left| {\dot{E}}_{b}^{i} \right| ^2}{2\eta _0} S_m \end{aligned} \end{aligned}$$

which through Eq. (28) leads to

47 $$\begin{aligned} P_{in} = \frac{1}{\eta _0}S_m. \end{aligned}$$

The absorbance then, can be estimated as the ratio of the absorbed power to the total input power, and hence,

48 $$\begin{aligned} A' := \frac{P_{d}+P_{m}}{P_{in}} = \frac{1}{4} \left| 1 + e^{j\Delta \phi } \right| ^2, \end{aligned}$$

which is equivalent to Eqs. (10) or (34), as expected.

### General slab model

**Figure 9:**
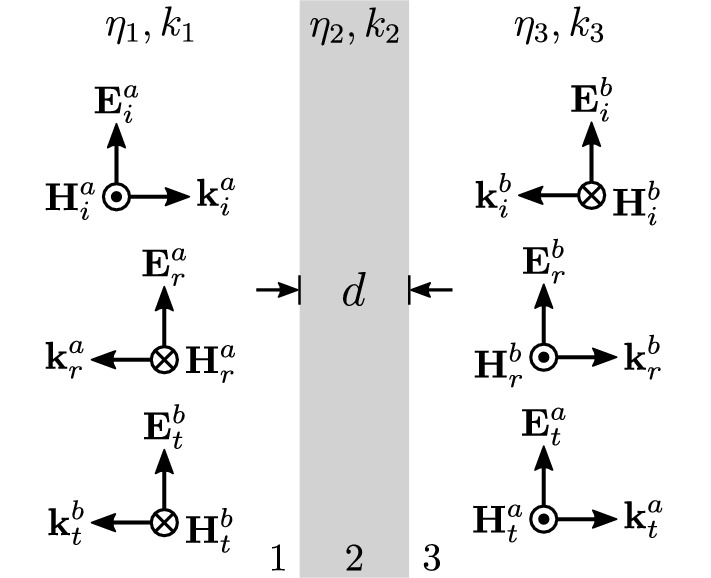
General slab model representation for the CPA of thickness *d*: the latter is normally illuminated by two plane waves, ${\mathbf {E}}_a^i$, ${\mathbf {E}}_b^i$.

Next, it is assumed that the zero thickness absorber is a homogeneous and isotropic slab of thickness *d* with relative permittivity and permeability of $\varepsilon _r$ and $\mu _r$, respectively. It is also assumed that the medium presents losses for both the electric and the magnetic field, and thus, in general the $\varepsilon _r$, $\mu _r$ could be complex values. Figure 9 depicts two plane waves, ${\mathbf {E}}^a_i$ and ${\mathbf {E}}^b_i$, which impinge normally on the slab (medium 2) on either side. For the plane wave ${\mathbf {E}}^a_i$ the reflection and transmission coefficients are given by^47^

49a $$\begin{aligned} r^a&:= \frac{{\dot{E}}^a_r}{{\dot{E}}^a_i} = \frac{\rho _1 + \rho _2 e^{ - 2 j k_2 d }}{1 + \rho _1 \rho _2 e^{ - 2 j k_2 d} } \end{aligned}$$

49b $$\begin{aligned} t^a&:= \frac{{\dot{E}}^a_t}{{\dot{E}}^a_i} = \frac{\tau _1 \tau _2 e^{ - j k_2 d }}{1 + \rho _1 \rho _2 e^{ - 2 j k_2 d} }, \end{aligned}$$

where, $\rho _i$ and $\tau _i$ are

50a $$\begin{aligned} \rho _i&= \frac{\eta _{i+1} - \eta _{i}}{\eta _{i+1} + \eta _{i}} \end{aligned}$$

50b $$\begin{aligned} \tau _i&= 1 + \rho _i, \end{aligned}$$

with $i=1,2,3$ and $\eta _i$ is the intrinsic impedance for the medium 1, 2 and 3, respectively. For the plane wave ${\mathbf {E}}^b_i$ the reflection $r^b$ and transmission $r^b$ responses are calculated respectively.

Next, it is assumed that the slab is surrounded by air, and thus,

51a $$\begin{aligned} \eta _1&=\eta _3=\eta _0 \end{aligned}$$

51b $$\begin{aligned} k_1&= k_3 = k_0 , \end{aligned}$$

hence, Eqs. (49) and (50) through (51) result in

52a $$\begin{aligned} r^a&= \rho \frac{ 1 - v^2 }{1 - \rho ^2 v^2 } \end{aligned}$$

52b $$\begin{aligned} t^a&= v \frac{ 1 - \rho ^2 }{1 - \rho ^2 v^2 }, \end{aligned}$$

where,

53a $$\begin{aligned} \rho&= \frac{\eta _2-\eta _0}{\eta _2+\eta _0} \end{aligned}$$

53b $$\begin{aligned} v&= e^{-j k_2 d}. \end{aligned}$$

Assuming again that the medium 2 is reciprocal and symmetrical and given

54a $$\begin{aligned} \eta _2&= \eta _0 \sqrt{ \frac{\mu _r}{\varepsilon _r} } \end{aligned}$$

54b $$\begin{aligned} k_2&= k_0 \sqrt{ {\mu _r}{\varepsilon _r} } = \frac{2\pi }{\lambda } \sqrt{ {\mu _r}{\varepsilon _r} }. \end{aligned}$$

is,

55a $$\begin{aligned} r^b&= r^a = r \end{aligned}$$

55b $$\begin{aligned} t^b&= t^a = t, \end{aligned}$$

and thus,

56a $$\begin{aligned} r&= \frac{ \left( \frac{\mu _r}{\varepsilon _r} - 1\right) \sin \left( 2\pi \frac{d}{\lambda } \sqrt{ {\mu _r}{\varepsilon _r} } \right) }{ \left( \frac{\mu _r}{\varepsilon _r} + 1\right) \sin \left( 2\pi \frac{d}{\lambda } \sqrt{ {\mu _r}{\varepsilon _r} } \right) - j 2 \sqrt{ \frac{\mu _r}{\varepsilon _r} } \cos \left( 2\pi \frac{d}{\lambda } \sqrt{ {\mu _r}{\varepsilon _r} } \right) } \end{aligned}$$

56b $$\begin{aligned} t&= \frac{ j 2 \sqrt{ \frac{\mu _r}{\varepsilon _r} } }{ \left( \frac{\mu _r}{\varepsilon _r} + 1\right) \sin \left( 2\pi \frac{d}{\lambda } \sqrt{ {\mu _r}{\varepsilon _r} } \right) - j 2 \sqrt{ \frac{\mu _r}{\varepsilon _r} } \cos \left( 2\pi \frac{d}{\lambda } \sqrt{ {\mu _r}{\varepsilon _r} } \right) }. \end{aligned}$$

The joint absorbance for that case is estimated again through Eq. (27) by substituting Eqs. (28) and from (49) to (56) and it is given by

57a $$\begin{aligned} A&= 1 - \frac{1}{2} \, \frac{ \left| c_1 e^{j \Delta \phi } - c_2 \right| ^2 + \left| c_1 - c_2 e^{j \Delta \phi } \right| ^2 }{ \left| \left( 1- \sqrt{\frac{\mu _r}{\varepsilon _r}} \right) ^2 - e^{j 4\pi \frac{d}{\lambda } \sqrt{ {\mu _r}{\varepsilon _r} }} \left( 1+ \sqrt{\frac{\mu _r}{\varepsilon _r}} \right) ^2 \right| ^2 } \end{aligned}$$

where,

57b $$\begin{aligned} c_1&= 4 \sqrt{ \frac{\mu _r}{\varepsilon _r} } e^{j 2\pi \frac{d}{\lambda } \sqrt{ {\mu _r}{\varepsilon _r} }} \end{aligned}$$

57c $$\begin{aligned} c_2&= \left( e^{j 4\pi \frac{d}{\lambda } \sqrt{ {\mu _r}{\varepsilon _r} }} - 1 \right) \left( 1 - \frac{\mu _r}{\varepsilon _r} \right) \end{aligned}$$

In order the latter to be maximum and minimum when the incident waves are in- and out-phase, respectively, and based on the previously described ZT-model and on Eq. (9), it is apparent that must

58 $$\begin{aligned} \begin{bmatrix} r^a &{} t^a \\ t^b &{} r^b \end{bmatrix} = \begin{bmatrix} -1/2 &{} 1/2 \\ 1/2 &{} -1/2 \end{bmatrix}. \end{aligned}$$

No solutions exist of the system of equations on (58). Thus, we inset the variable *t* in order to find an approximate solution, where $t \rightarrow 0$ but not zero, and hence, Eq. (58) is transformed to,

59 $$\begin{aligned} \begin{bmatrix} r^a &{} t^a \\ t^b &{} r^b \end{bmatrix} = \begin{bmatrix} -1/2+t &{} 1/2-t \\ 1/2-t &{} -1/2+t \end{bmatrix}, \end{aligned}$$

with solutions

60a $$\begin{aligned} \rho&= \frac{1 \pm 2\sqrt{t\left( 1-t \right) }}{2t-1} \end{aligned}$$

60b $$\begin{aligned} v&= \frac{ -1 \mp 2\sqrt{t\left( 1-t \right) }}{2t-1} \end{aligned}$$

In that case the joint absorbance is

61 $$\begin{aligned} A = 1 - \left( \frac{1}{2}-t \right) ^2 \left| 1 - e^{j\Delta \phi } \right| ^2 \end{aligned}$$

and thus, the system presents $A=1$ when the waves are in-phase regardless *t*, however for the out-phase case the system does not present zero absorbance but $A=1-4(1/2-t)^2$.

The parameters $\eta _2,~k_2$ are given based on Eq. (53)

62a $$\begin{aligned} \eta _2&= \eta _0 \frac{1+\rho }{1-\rho } \end{aligned}$$

62b $$\begin{aligned} k_2&= \frac{1}{d} \left( 2 \pi c + j \log v \right) , \end{aligned}$$

where $c \in {\mathbb {Z}}$. and via equation (54),

63a $$\begin{aligned} \varepsilon _r&= \pm \frac{k_2}{k_0} \frac{\eta _0}{\eta _2} \end{aligned}$$

63b $$\begin{aligned} \mu _r&= \pm \frac{k_2}{k_0} \frac{\eta _2}{\eta _0}. \end{aligned}$$

and through Eq. (60) is

64a $$\begin{aligned} \varepsilon _r&= \pm \frac{\lambda }{d} \left( c + j \frac{\log v }{2 \pi } \right) \frac{1-\rho }{1+\rho } \end{aligned}$$

64b $$\begin{aligned} \mu _r&= \pm \frac{\lambda }{d} \left( c + j \frac{\log v }{2 \pi } \right) \frac{1+\rho }{1-\rho }. \end{aligned}$$

and hence, the relative permittivity and permeability of the coherent absorber with thickness of *d* ends up being a function of its thickness and the operating frequency. For example, at 10 GHz, assuming $d= 3~{\mathrm {mm}} \approx \lambda _{10\,{\mathrm {GHz}}}/10$, $c=1$, $t=0.01$ and choosing the second set of solutions of Eqs. (60) is via equation (62)

65 $$\begin{aligned} \eta _2 = 37.89~{\mathrm {\Omega }},~ k_2 = 2096 - j 68.28~{\mathrm {rad/m}} \end{aligned}$$

and via Eq. (64),

66 $$\begin{aligned} \varepsilon _r = 99.5 - j 3.19 , \mu _r = 1 - j 0.032. \end{aligned}$$

**Figure 10:**
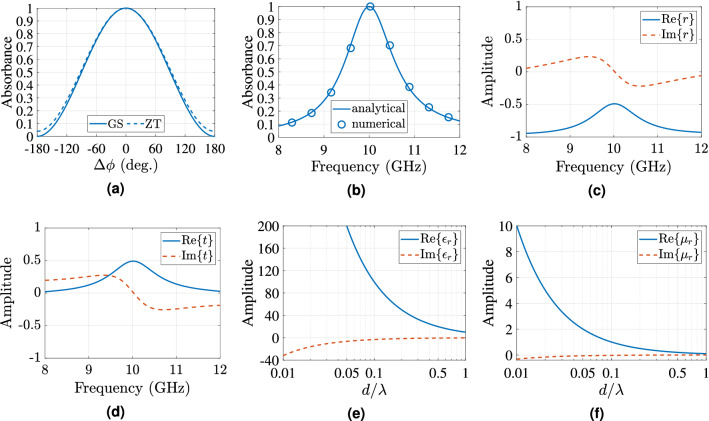
The theoretical joint absorbance versus $\Delta \phi$ at 10 GHz (**a**) and versus frequency for in-phase incident signals, i.e., $\Delta \phi = 0{^{\circ }}$ is depicted for the GS- and ZT-model, based on Eqs. (57) and (10), respectively. The general slab was also tested thought simulation using the frequency CST electromagnetic solver and the simulated absorbance versus frequency is also depicted alongside with the theoretical results (**b**): a very good agreement is observed. Specifically, the slab was modelled as a homogeneous dielectric slab with dielectric permittivity, permeability given in Eq. (66). Also, the reflection (**c**) and transmission (**d**) coefficients versus frequency based on Eq. (56) is depicted. For all the previous cases please note for the slab that, $\varepsilon _r=99.5-j3.19$, $\mu _r=1-j0.032$ and has thickness of $3~{\mathrm {mm}} \approx \lambda _{10\,{\mathrm {GHz}}}/10$. It is also depicted the dielectric permittivity (**e**) and permeability (**f**) versus thickness *d* over $\lambda$ for the GS-model on Eq. (64) and for $t=0.01$.

Figure 10 depicts the theoretical analysis of the general slab (GS) model. Specifically, in Fig. 10a is depicted the joint absorbance for normal incidence based on the general slab model (estimated through equations on (57) and (66)) versus the ZT-model (estimated through Eq. (10)). The models for this case agree very well. The main difference occurs for the out-of-phase case, where the GS-model predicts non-zero absorbance of 0.0396, based on (61). Next, the GS-model absorbance is tested versus frequency. Again, Eqs. (57) and (66) were applied and the result is depicted in Fig. 10b: it is evident that the GS-model significantly lags in terms of operation bandwidth (i.e., $A \ge 0.9$ for 9.78–10.22 GHz, hence presents fractional bandwidth of only $4.4\%$) compared to the frequency independent ZT-model. The GS-model’s joint absorbance versus frequency was also numerically tested and the results are also depicted in Fig. 10b: a good agreement is observed between the numerical and analytical results. For the numerical simulation the electromagnetic solver of the CST suite was again used and the slab was modelled as a homogeneous medium with $\varepsilon _r=99.5 - j3.19$ and $\mu _r=1-j0.032$. Fig. 10c,d depict the theoretical results for the reflection and transmission coefficients based on Eq. (56), respectively, versus frequency applying (Eq. 66). Please note that, for all the previous cases the thickness of the slab was assumed $d= 3~{\mathrm {mm}} \approx \lambda _{10\,{\mathrm {GHz}}}/10$. Also, Fig. 10e,f depict the dielectric permittivity and permeability versus the thickness of the slab over lambda, as estimated on Eq. (64), through Eq. (60) for $t=0.01$.

The above analysis proved that the reconfigurable absorber of the ZT-model presents superior performance in terms of frequency bandwidth, design process and complexity compared to the general slab behaviour, as expected, since the slab has non-zero thickness.

### Cross-polarized reflections

It is noted that, based on the theoretical analysis of the zero thickness and general slab model, for the proposed CPA the reflected and transmitted plane wave has the same polarisation with the incident plane wave: for example, a TE (or TM)-polarised incident plane wave will result in TE (or TM)-polarised reflected and transmitted plane wave. Thus, the cross-polarised reflection and transmission coefficients are expected to be zero, as mathematically described by Eq. (22) and schematically represented in Fig. 5a. The reason is that, the geometry of the proposed CPA is completely, isotropic, linear, passive, homogeneous, uniform and symmetrical, with no structures (e.g., chiral geometries) which should create cross-polarized reflections from the structure. For this reason, we have not presented coefficients of this type.

### Experimental results

Based on the ZT-model, a screen with surface resistance of $\eta _0/2$ was fabricated through conventional inkjet printing technology, using an Epson C88+, and then it was measured. For our tests, the resistivity loaded screen was printed on a coated polyethylene terephthalate (PET) sheet ($\varepsilon _r=2.95$, $\tan \delta =0.025$) of thickness 0.14 mm provided by Novacentrix^48^. It is noted that the sheet impedance was measured (as it will be shown) at the microwave region and specifically at frequency 10 GHz. In that case, the PET has thickness of only $~\lambda /215$ and its impact on the absorber’s performance is very low. Specifically, theoretical and numerical tests were performed for the resistivity loaded sheet without (ZT-model) and with the PET (realistic scenario) and the calculated error for the estimated *S* parameters (ZT-model versus realistic scenario) was lower than $0.33\%$ for the frequency region 8–18 GHz (maximum error at 18 GHz), and thus, the estimated absorbance remained unchanged.

**Figure 11:**
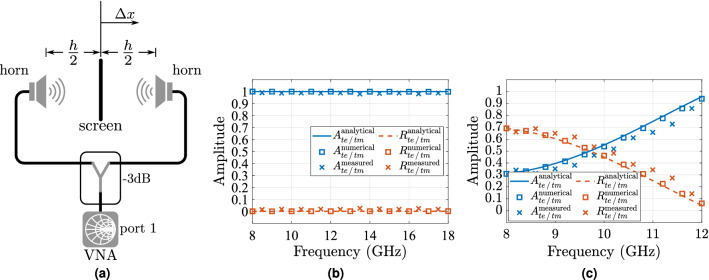
Measured vs analytical vs numerical results of the coherent absorber. (**a**) Setup measurement test for the reflection coefficient of the resistively loaded screen. Signals are in phase (i.e., $\Delta \phi = 0{^{\circ }}$) when $\Delta x = 0$ mm. The reflectance and corresponding absorbance of a resistively loaded screen of $60\pi \ \Omega$/sq (i.e., $\eta _0/2$) surface resistance printed on a PET sheet, which is normally illuminated by TE- and TM-polarised incident incident waves, which are in-phase, i.e., $\Delta x = 0$ mm, (**b**) and out-of-phase, i.e., $\Delta x = 20$ mm or equivalently $\Delta \phi = 240{^{\circ }}$ (**c**).

The measurement with coherent illumination was performed by placing two sets of horn antenna pairs (8–12 GHz and 12–18 GHz) at a distance of $h/2=16$ mm from the sample, i.e., $\Delta x = 0$ mm (Fig. 11a), and the comparison between the measured, analytical and numerical results for reflection and absorption are shown in Fig. 11b,c, where the frequency range was limited by the available equipment for the experiment. The measured results show near perfect absorption (i.e., $100\%$) of the signal for coherent illumination for the tested frequency region of 8—18 GHz, and an excellent agreement with the numerical and analytical predictions. Please note that, the numerical results agree very well with the theoretical ZT-model.

Additionally, to demonstrate the CPA absorbance reconfigurability, a measurement was taken when the sample was not placed midway between the two horn antennas, i.e., different phase illumination, due to an offset $\Delta x = 22$ mm. Here, although without a control signal as shown in Fig. 1c, we can adjust $\Delta \phi$ by altering the physical location of the antennas. For example, at 10 GHz for a $\Delta x=20$ mm it is $\Delta \phi =240{^{\circ }}$, at 12 GHz it is $\Delta \phi = 288{^{\circ }}$, etc. For this experiment, we have chosen to work only with the pair of X-Band horn antennas (8–12 GHz) as creating the same electric distance between the second pair, 12–18 GHz, was not possible given the available equipment, as it will be explained in the measurement set-up section. This can be avoided by using a set of wideband horn antennas covering the desired bandwidth, which was not available in our laboratory. However, this does not prevents one from noticing the difference in absorption throughout the measured bandwidth as depicted in Fig. 11c, and good similarity with the numerical and analytical predictions.

## Discussion

In this paper, a comprehensive set of analyses were used to explain the working mechanism of reconfigurable passive CPA. We mathematically demonstrated that a resistive sheet with a surface impedance of $\eta _0/2$ satisfies the maximum power transfer condition and therefore absorbs coherent electromagnetic waves impinging on both sides of the sheet, which can also be verified via theoretical ZT- and GS-models. Moreover, the GS-model is compared to the ZT-model and the results are in favour of the latter in terms of frequency bandwidth, design and complexity. The models are verified via full electromagnetic analysis and in experiments, performed in a semi-anechoic chamber: simulated and measured results are in good agreement with the proposed theoretical models. The fabricated CPA demonstrates a near perfect absorption within the measured frequency range for coherent waves and that the absorbance can be tuned by outphasing the signals to achieve the desired value of reflection coefficient on the surface.

## Methods

### Fabrication process and characterization

The resistivity of the screen is achieved by using the electrically conductive nano-Ag ink Metalon$^{\mathrm {TM}}$ JS-B25P45^49^: the latter, dissolved in a aqueous inkjet vehicle (ethylene glycol and glycerine) and the mixture is stored in an empty inkjet cartridge to be used by the printer, which in our case, is the conventional inkjet printer Epson Stylus C88+. Please note that, the latter is a 4-colour inkjet printer and the conductive ink mixture was placed in the cartridge which corresponds to the cyan colour. The requested surface impedance is achieved by adjusting two parameters: first is the volume ratio of the conductive ink to the aqueous solution, which in our case was set to $1_{\mathrm {ink}}:6_{\mathrm {aq}}$(volume) based on the analysis in^21^, and second is the grey scale (through the RGB-code) on the printer. In general, it is known that RGB (0, 0, 0) results black colour (maximum spatial density of ink), RGB (255, 255, 255) results white (lack of ink) and RGB values in between results gray-scale^21^. The same concept is applied to our case where, the cyan scale, in which RGB (0, 255, 255) results in $100\%$ cyan, i.e., results maximum spatial density of ink, while tends to white, i.e., lack of ink, as the first value increases towards 255. On the other hand, as the density of the ink increases, the spatial density of the resistive droplets also increases, and thus, more overlap between the droplets occurs resulting in the low resistivity (i.e., based on the setup which was used in our case RGB (0, 255, 255) and (255, 255, 255) results the minimum and maximum resistivity for the current ink-to-water ratio). The whole fabrication procedure is also explained in^18,21^: based on this work and on validation measurements^50^ in order to achieve surface resistance of $60\pi$ $\Omega /{\mathrm {sq}}$ the volume ink-to-water ratio was fixed at 1 : 6, as mentioned above, and the RGB code at (75, 255, 255).

**Figure 12:**
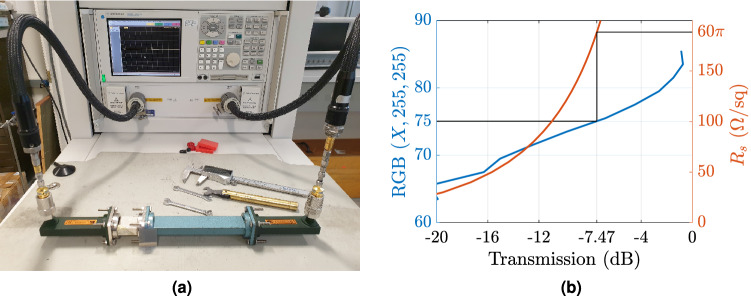
Waveguide characterization of the surface resistance to determine the correct RGB for the resistive sheet (**a**). Characterization of the resistive samples (**b**). Many resistively loaded sheets were printed and placed in the middle of a X-band waveguide and the transmission coefficient was measured and depicted at 10 GHz. Each of these samples corresponds to a specific RGB code (left axis). Additionally, the same scenario (a resistive sheet printed on a PET substrate, placed in the middle of the waveguide) was simulated: now, the resistivity varies (right axis) and again the transmission coefficient was estimated. Resistivity of $60\pi \ \Omega$/sq (i.e., $\eta _0/2$) results to simulated/measured transmission coefficient of $-7.47$ dB, which corresponds to RGB code of (75, 255, 255).

Figure 12a depicts the experiment setup used to determine the RGB code, which corresponds to $60\pi$ $\Omega /{\mathrm {sq}}$ surface resistance. Specifically, the transmission coefficient was measured using an X-Band rectangular waveguide. The samples, which are square shaped patches printed on the PET substrate^50^, were exactly placed in the middle of the waveguide and the RGB code was ranging $(60-80,255,255)$: we printed several samples with different RGB code in order to cover all this region. On the other hand, the same scenario was also simulated using the CST Microwave Studio: the ohmic’s sheet resistivity ranged 0–200 $\Omega$/sq and the transmission coefficient was estimated again. The simulation setup consisted of two rectangular waveguides of identical dimensions used for the measurements, each fed by a waveguide port. The 0.14 mm thick PET material with a zero-thickness layer of an Ohmic sheet ($R_s=60\pi$ $\Omega$/sq) was placed between the two waveguides, and the transmission coefficient (shown in Fig. 12b was obtained for its fundamental propagation mode. Thus, simulated resistivity of $60\pi$ $\Omega$/sq (i.e., $\eta _0/2$) results $-7.47$ dB transmission coefficient, which in turn, corresponds to RGB code of (75, 255, 255) , as depicted in Fig. 12b.

### Measurement set-up

**Figure 13:**
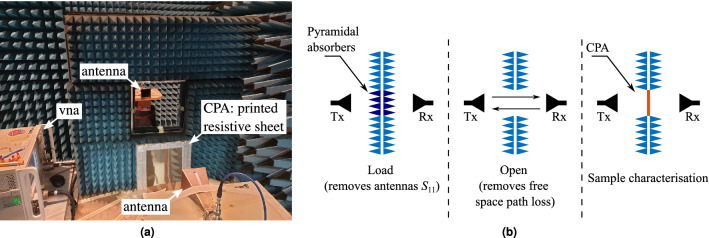
Measurement set-up. The aluminium tape around the sheet prevents the energy from escaping from the sides of the square window (**a**). Calibration steps followed for the CPA measurements (**b**).

To verify the theoretical model, a $210\ {\mathrm {mm}}\times 270\ {\mathrm {mm}}$ sheet was printed using RGB code (75, 255, 255) on a letter size PET substrate (0.14 mm thick). The measurements were performed in a semi-anechoic chamber using two pairs of horn antennas (8–12 GHz and 12–18 GHz) in which an absorbing screen with a $30\ {\mathrm {cm}}\times 30\ {\mathrm {cm}}$ window was used to hold the sample which was fixed on an Rohacell ($\varepsilon _r=1.05$, $\tan \delta <0.00017$) foam to ensure that the sample was flat during the experiment, as shown in Fig. 13a.

The measurement was performed as depicted in Fig. 11a, in which a Macom 2090-6210 Wilkinson power divider was used to equally split the energy from port 1 of the PNA to two phase matched precision SMA cables carrying the energy to the horn antennas. It is worth noting that although the theoretical analysis demonstrated a frequency independent absorber, our measurements were limited by the facilities in the laboratory, such as antennas and the power divider.

An experiment performed with the set-up as in Fig. 11a, requires a two steps calibration process, prior the measurement to characterise the sample, as illustrated in Fig. 13b. The first step consists in placing the antennas equally spaced from a pyramidal absorber, which ensures a suppression of at least 50 dB at this frequency range. As the absorber effectively decouples the antennas from the environment in the semi-anechoic chamber, the measured $S_{11}$ refers to the antenna’s own reflection coefficient, which needs to be eliminated from the characterisation process to ensure that the measured $S_{11}$ from Fig. 11a belongs to the CPA. The next step removes the path loss contribution to the measurement by removing any obstacle between the antennas and obtaining its reflection coefficient. Let $S_{11p}$, $S_{11o}$ and $S_{11c}$ represent the reflection coefficient from the pyramidal absorber, *open* space and calibration, respectively. The reflection coefficient of the CPA, $S_{11cpa}$ will be given by:

67a $$\begin{aligned} S_{11c}&= S_{11o}-S_{11p} , \end{aligned}$$

67b $$\begin{aligned} S_{11cpa}&= S_{11meas}-S_{11c}, \end{aligned}$$

where $S_{11meas}$ refers to the measured reflection coefficient obtained from the CPA before normalisation.
